# Exploring the barriers and facilitators to safer hair product purchasing and use among Black women in the greater Boston area through photovoice in the RESTYLE study

**DOI:** 10.3389/fpubh.2025.1513671

**Published:** 2025-08-18

**Authors:** Marissa Chan, Marlee R. Quinn, Jackeline Morales, Cynthia Jones, Kalya Murray, Gary Adamkiewicz, Leopoldo J. Cabassa, Tamarra James-Todd

**Affiliations:** ^1^Department of Environmental Health, Harvard T.H. Chan School of Public Health, Boston, MA, United States; ^2^Department of Environmental Health, Boston University School of Public Health, Boston, MA, United States; ^3^Union Capital Boston, Boston, MA, United States; ^4^Bethel Institute for Community Development, Boston, MA, United States; ^5^Center for Mental Health Services Research, Washington University in St. Louis, St. Louis, MO, United States; ^6^George Warren Brown School of Social Work, Washington University in St. Louis, St. Louis, MO, United States; ^7^Department of Epidemiology, Harvard T.H. Chan School of Public Health, Boston, MA, United States

**Keywords:** photovoice, hair products, community-based participatory research, facilitators, barriers, endocrine disrupting chemicals

## Abstract

**Introduction:**

Racial/ethnic differences in personal care product (PCP) use, including hair products, are well-documented in the United States (US). Black women are more highly exposed to endocrine disrupting chemicals in PCPs compared to other racial/ethnic groups. We identified barriers and facilitators to safer hair product purchasing and use in the greater Boston, Massachusetts area.

**Methods:**

Twenty-one Black women were recruited through community organizations to participate in photovoice as a part of the Retail Environment and Hair Styling Exposure (RESTYLE) study. Participants as co-researchers were trained in photography/PCP safety preceding a focus group. The co-researchers took photographs based on a prompt and then engaged in an interview and a focus group to discuss the photos. The co-researchers participated in another round of photography, interviews, and focus groups based on a second co-developed prompt. All study activities were virtual. Deductive and inductive approaches were used to code and analyze the data using NVIVO Version R1.

**Results:**

Five themes related to barriers were identified (e.g., unsafe ingredients allowed in US products and differences in access to safer hair products). Two concepts underlying the barriers were distrust in government/large corporations and individual burden/responsibility to shop for safer products. The two themes related to facilitators were going back to our cultural and community roots and raising individual knowledge and public awareness for action.

**Discussion:**

Photovoice is a powerful community-based participatory method rooted in community experiences. Several barriers and facilitators to safer hair product purchasing and use were identified among the co-researchers’ lived experiences that can inform future research and interventions.

## Introduction

Personal care products are an underregulated market in the United States (US), and the Food and Drug Administration (FDA) has only prohibited or restricted the inclusion of 11 ingredients in its history (1). Thus, personal care products, such as hair products, are found to contain a variety of chemicals of concern including endocrine disrupting chemicals (EDCs)—external substances, both natural and man-made, that can interfere with the body’s hormone system through various mechanisms (2). Some common personal care product-associated EDCs include phthalates, parabens, and triclosan (3). While exposure to EDCs is pervasive, inequities are reported with Black women being more highly exposed compared to other racial/ethnic groups (4, 5). Differences in product use patterns may contribute to inequities in exposure to EDCs (6, 7). In terms of hair products, Black women are found to be more likely to use certain leave-in products (leave-in conditioner, hair oil) and chemical hair relaxers (6–8) and less likely to use certain rinse-off hair products (shampoo, conditioner) compared to other racial/ethnic groups (9, 10). Furthermore, these same hair products commonly used by Black women are reported to be hormonally active and contain EDCs (11, 12). The more frequent use of certain personal care products has translated to higher chemical exposures, as well as adverse health outcomes ranging from higher risk of earlier age at menarche, low birth weight, and ovarian cancer in Black women (9, 13, 14). Thus, previous exposure assessment and epidemiological studies have consistently presented evidence that Black women are both more highly exposed to personal care product associated EDCs and at an increased risk of health outcomes compared to other racial/ethnic groups in the US.

Community-based participatory research (CBPR) is an innovative approach that aims to break down power dynamics and democratize knowledge through equal and mutually beneficial partnerships (15). CBPR focused on health equity is centered around the co-creation of knowledge and the identification of solutions to reduce health inequities (16). Several community-based studies have explored the drivers of personal care product use among diverse communities and reported factors from the individual level to the societal level contributing to product use (7, 17–19). Two of these studies used qualitative methods to explore different questions regarding hair product use among individuals of color and discussed the role of social pressures (i.e., beauty standards) contributing to hair product use patterns (18, 19). While these studies are important to understand the socio-contextual factors relevant to personal care product use, they do not specifically identify the barriers and facilitators to safer product use—an essential step towards developing interventions and solutions.

Through photovoice, a community-based participatory qualitative method that uses photographs and the stories behind these visuals to help identify community issues and resources (20, 21), we aimed to explore the common experiences surrounding the barriers and facilitators to safer hair product purchasing and use among Black women in the greater Boston, Massachusetts (MA) area.

## Materials and methods

### RESTYLE study

The Retail Environment and Hair Styling Exposure (RESTYLE) Study aims to explore the community and neighborhood-level drivers of hair product availability and use patterns and to identify potential community-based interventions in the greater Boston, MA area. RESTYLE is a mixed-methods community-based study that recruited individuals from 2023 to 2024. Individuals who participated in the photovoice project were recruited from June 2023 to September 2023. The following analysis reports on the results of the data collected using photovoice, a participatory qualitative method using photography, individual interviews, and focus groups (21, 22). All co-researchers provided informed consent, and the study was approved by the Harvard Longwood Campus Institutional Review Board.

The RESTYLE Study is grounded in the National Institute of Minority Health and Health Disparities (NIMHD) Research framework which conceptualizes factors relevant to health at different levels of influence spanning from individual to societal and through different domains of influence across the life course including the built and sociocultural environments (23). This framework guided our research questions, coding of interviews and focus groups, and analysis/interpretation of the qualitative data.

### Community partners and recruitment

We partnered with several Boston-based organizations and institutions: Bethel AME Church, Union Capital Boston, Resilient Sisterhood Project, Southern Jamaica Plain Health Center, and Comics in Color. These community partners were a combination of new and longstanding partnerships. For additional details on our community partners see Supplementary Table S1. These partnerships informed our study design and facilitated the recruitment of Black women in the greater Boston area to join RESTYLE.

Recruitment occurred through a multipronged approach. Our community partners reached out to their members through announcements after church services, social media posts, printed flyers, direct outreach to specific members who may be interested, and/or emails to listservs. The study staff also engaged in direct community outreach through tabling at events and presenting virtually at a community meeting.

### Study population

Twenty-five co-researchers were enrolled in RESTYLE photovoice. The term co-researchers was used instead of participants based on our use of an anticolonial approach to photovoice (24) (described in the *Photovoice section*) that is rooted in respect and equal partnerships. Individuals were included if they self-identified as Black or of African descent, female or femme-identifying, 18 years or older, and were proficient in English. After enrolling in the study, each co-researcher completed a brief sociodemographic survey. All enrolled co-researchers preferred virtual study activities (focus groups and interviews via Zoom or phone calls) to in-person. Four co-researchers were lost to follow up—one for health reasons and the remaining three for unknown reasons. In total, 21 co-researchers were involved in the entire photovoice process from August 2023 to December 2023.

### Photovoice

Photovoice is a participatory qualitative research method that is rooted in the participants as co-researchers (24) reflecting upon their community to identify resources and challenges and begin to develop solutions (21). The three original goals of photovoice are (1) to enable people to record and reflect on their community’s strengths and concerns, (2) to promote critical dialog and knowledge about important issues through small group discussions of photographs, and (3) to reach policymakers (21). Empowering co-researchers through the participatory research process is also a goal of this method (21). Supplementary Figure 1 shows the photovoice process.

We utilized an anticolonialist approach to photovoice that aims to break down power dynamics and views those involved as actively engaging and directing the research process instead of participating (24). Specifically, we implemented the use of the term co-researchers instead of participants, allowed the co-researchers to choose a pseudonym or remain anonymous, and relied on verbatim and longer quotes when possible to ensure the co-researchers stories were directly shared instead of summaries which may unintentionally or intentionally modify the meaning.

For the photovoice process, we first hosted a combined training and focus group. The 30 to 45-min training consisted of (1) best practices and ethical considerations in photography, guided by Wang and Redwood Jones (25), and (2) an introduction to personal care product safety. The training on personal care product safety consisted of an overview of the US regulatory landscape, sources of EDCs, common personal care product EDCs, and potential associated health outcomes. Following the training, we hosted a 30 to 45-min exploratory focus group where the study staff transitioned from the role of trainer to focus group facilitator. This focus group allowed the co-researchers to reflect on their hair product decision-making processes which may have guided their photography. A portion of this focus group informed the choices of keywords used in the photography prompts (i.e., facilitator, barrier, support, prevent, natural, safe).

Following the training/focus group, the co-researchers were asked to take photos of *anything that prevents or supports safer or more natural hair product purchasing and use* (prompt 1) over the next 2 weeks. The choice of the words safer or more natural depended on the groups’ preferences earlier in the session. The co-researchers had the choice of using their phone or a disposable camera. Study staff checked in via text or phone call at the halfway point. Next, brief photo-elicitation interviews (10 to 20 min) were conducted, guided by questions from Cabassa et al. (22), to assist the co-researchers in selecting one photo to share in the focus group. Following the interview, the co-researchers provided titles and captions describing their experience and connection to the theme. We then hosted a second set of focus groups (60 to 70 min) guided by modified S. H. O. W. E. D questions (21). S. H. O. W. E. D questions include: what do you *See*? What’s *Happening*? How does this relate to *Our* lives? *Why* do these issues or strengths *Exist*? What can we *Do* about this issue? At the end of the second focus group, the main ideas were summarized, and a new prompt was identified through group consensus. This process of engaging in photography, a photo-elicitation interview, and a focus group repeated with the new theme (Supplementary Figure S1).

Four groups of co-researchers went through the photovoice process from August 2023 to December 2023 (Supplementary Figure S1). Each group had between four and seven co-researchers. One co-researcher who had learning differences transitioned from focus groups to individual interviews after the first training based on the focus group dynamics and to ensure she had the space to express her experiences. Two co-researchers from Group 4 (G4) attended a make-up focus group 3 due to family emergencies. In total, each co-researcher participated in three focus groups and two interviews. Co-researchers were compensated for each interview and focus group they were a part of via gift cards. Meal gift cards were also provided for each focus group.

### Data analysis

Summary statistics were conducted to describe the sociodemographic characteristics of our co-researchers. All interviews and focus groups were recorded, transcribed, and coded (with NVIVO version R1). Zoom chats were also transcribed and included in the transcripts. We used a combination of pile sorting techniques from Bernard 2017 and analytic techniques from Crabtree and Miller 1999 (organizing, connecting, and corroborating) to analyze the interview and focus group transcripts (26, 27).

**Figure 1:**
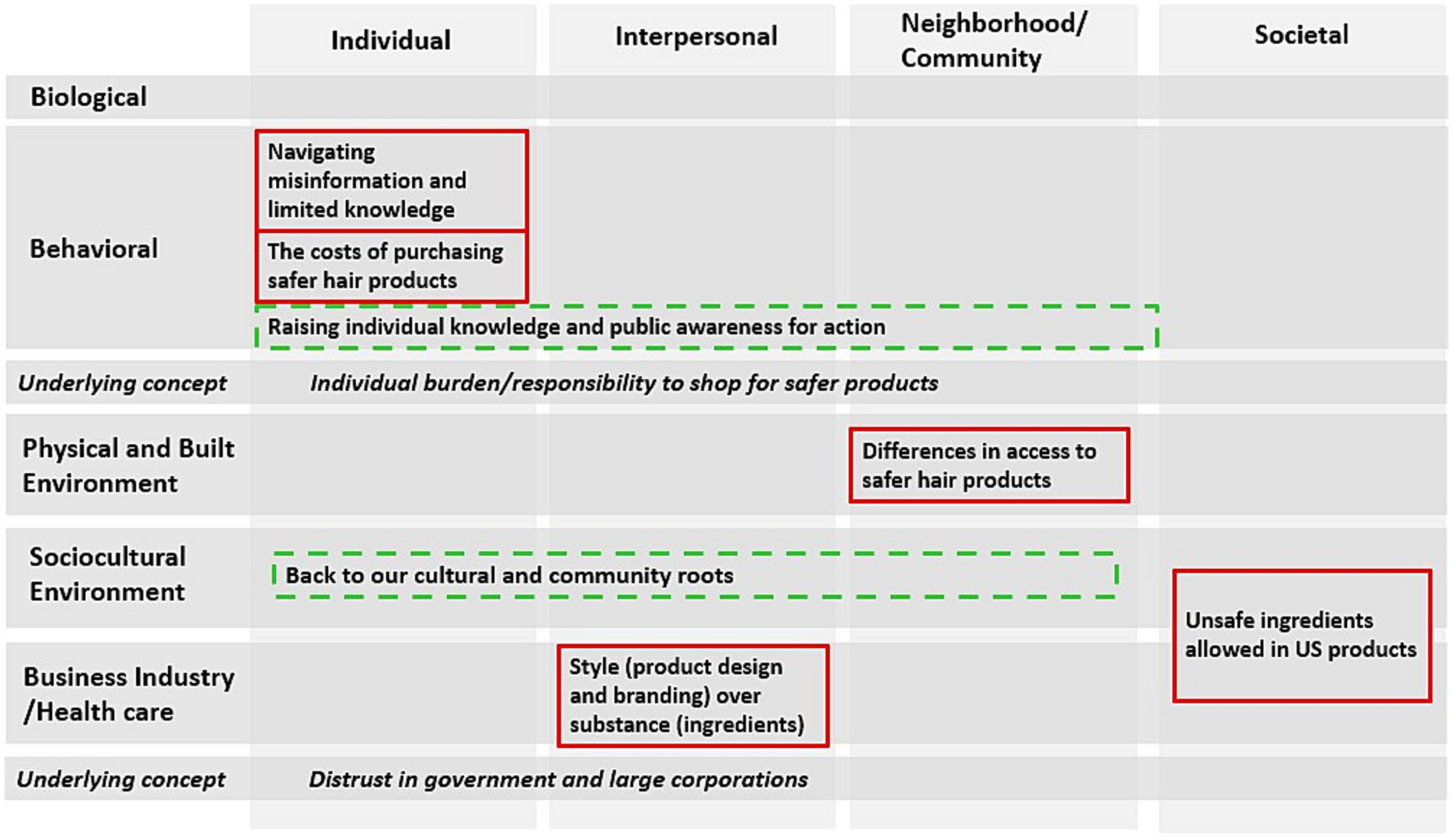
Figure of the barriers, facilitators, and underlying concepts identified using a modified National Institute of Minority Health and Health Disparities Research Framework (this figure has the same levels and domains of influence with the addition of businesses to the healthcare industry domain); the barriers (solid red line) included: (1) unsafe ingredients allowed in US products, (2) differences in access to safer hair products, (3) style (product design and branding) over substance (ingredients), (4) the costs of purchasing safer hair products, and (5) navigating misinformation and limited knowledge. The facilitators (dashed green line) included: (1) back to our cultural and community roots and (2) raising individual knowledge and public awareness for action. The underlying concepts included: (1) Distrust in government and large corporations and (2) individual burden/responsibility to shop for safer products.

We used pile sorting to develop codes for the interviews, as done by previous photovoice research (22). In brief, this technique consists of placing the photographs and transcripts from the photo-elicitation interviews into piles based on similarities (22, 26, 28). The resulting codes were added to the codebook—some codes include word of mouth, branding, and resources. For the focus group transcripts, deductive (confirmatory) and inductive (exploratory) approaches to coding were taken, where both codes were developed *a priori* based on the literature and our theoretical framework (NIMHD Research framework) as well as identified from the transcripts through open coding. For example, based on previous research and the NIMHD framework (individual level and behavioral domain of influence) “cost” was selected as a code. Additionally, from the transcripts “word of mouth” was identified as a code. Specifically, an editing organizing style was used where we coded information based on categorizing the text in ways relevant to our research question (27). Through the “connecting” phase we discovered patterns and eventually themes between the codes. Themes arose from analyzing the codes identified from the qualitative photovoice data and represent underlying patterns between codes that explain a phenomenon (27, 29). This process consisted of generating code queries in NVIVO, tracking common ideas and patterns, identifying connections between codes, and generating visual diagrams of the codes to aid in identifying the connections. Additionally, we developed a diagram that presents our themes and shows the connections to the domains and levels of influence from the NIMHD research framework (Figure 1). Finally, through “corroborating”—we confirmed the patterns and themes by re-reviewing the transcripts and visual diagrams to identify any evidence that may not support the themes. Any notable but not significant disconfirming evidence (significant indicating potentially an incorrect theme) was flagged to discuss in the results to ensure the nuances in experiences were appropriately discussed. As a note, the use of “our” in the results refers to the co-researchers and their experiences and perceptions.

To ensure trustworthiness in our analysis, we used the specific quality criteria of credibility and dependability/confirmability (30). In terms of credibility, we used the strategies of prolonged engagement through our multiple interviews, focus groups, and check-in meetings to build trust and gain additional insights. Furthermore, we used investigator triangulation for five of the 11 focus groups conducted where two researchers (MC and JM) coded the same text and points of contention were reviewed. For dependability/confirmability, we took extensive notes during the entire process from transcription to coding and analyzing the data to develop an audit trail.

## Results

Table 1 presents the sociodemographic characteristics of the co-researchers. The co-researchers’ average age (mean, standard deviation) was 48.2 years (11.3). All co-researchers self-identified as of African descent—with the majority identifying as Black/African American (81%) and born in the US (85.7%). Educational attainment and income levels varied, with the highest percentage of co-researchers attending some college or with an associate degree (42.8%). Most co-researchers had Medicaid for insurance (47.6%). Sixty percent lived in the city of Boston.

**Table 1:** Self-reported co-researcher characteristics.

Characteristic	Mean (SD) or count (%)
Mean age (Standard Deviation) (*n* = 19)^a^	48.2 years (11.3)
Self-identified Race/ethnicity (*n* = 21)	
Black/African American	17 (81)
Black-Caribbean	1 (4.8)
Black-Hispanic-Central American	1 (4.8)
Hispanic-Puerto Rican	1 (4.8)
Hispanic-Dominican	1 (4.8)
Country of Birth (*n* = 21)	
US	18 (85.7)
Dominican Republic	1 (4.8)
Honduras	1 (4.8)
Trinidad	1 (4.8)
Educational Attainment (*n* = 21)	
< 12th grade	1 (4.8)
Graduated from high school/ GED	4 (19.0)
Some College/ Associate degree	9 (42.8)
Graduated from College	2 (9.5)
Graduate degree	5 (23.8)
Household Income (*n* = 19)	
≤$9,999 per year	4 (21.1)
$10,000–$24,999 per year	3 (15.8)
$25,000–$49,999 per year	4 (21.1)
$50,000–$74,999 per year	3 (15.8)
$75,000–$99,999 per year	2 (10.5)
≥$100,000 per year	3 (15.8)
Insurance (*n* = 21)	
Self-pay	0 (0)
Private Insurance/HMO	8 (38.1)
Medicaid/SSI/Mass Health	10 (47.6)
None	2 (9.5)
Unsure	1 (4.8)
Location (*n* = 20)	
City of Boston	12 (60)
The greater Boston area (outside the city of Boston)	8 (40)

a The co-researchers were not required to respond to every question in the demographic survey; sample sizes for each characteristic ranged from 19 to 21.

Each co-researcher selected two photos through the photovoice process (except for two who selected three photos). Thus, 44 photos (a combination of those taken by the co-researchers and screenshots from internet searches) were included. All 44 photos and captions can be viewed at the virtual photo gallery (www.restylestudy.com). The prompts developed by each group of co-researchers for the second round of photography were: (1) exploring what ingredients are in hair products and how they may impact our health, (2) exploring the role of businesses and suppliers in influencing natural and safer hair product purchasing and use, (3) exploring and researching natural ingredients that are good for our health to incorporate into our hair care routine, and (4) exploring the role of do-it-yourself (DIY) approaches or making your own hair products that are safer and good for your health.

### Themes

Table 2 presents the themes related to the barriers (5 themes) and facilitators (2 themes) to safer hair product purchasing and use among the RESTYLE co-researchers. Two concepts underlying the barriers were also highlighted. Figure 1 presents the themes identified in the context of the NIMHD research framework. We utilized the same levels and domains of influence except for the addition of business to the healthcare industry domain (added to incorporate a relevant industry to personal care products into the framework).

**Table 2:** Summary of themes and concepts related to barriers and facilitators to safer hair product purchasing and use.

Theme/Concept	Summary of results
Barriers	
Unsafe ingredients allowed in US products	Lack of government regulations regarding ingredients in products Ingredients of concern include endocrine disruptors and preservatives
Style (Product Design and Branding) Over Substance (Ingredients)	Products designed to attract consumers Ingredients labels are small and difficult to read Certain words only used for branding
Differences in access to safer hair products	Differences in products available between neighborhoods/communities Barriers to purchasing products or reading labels in store (plexiglass barrier or theft sensor on label)
Navigating misinformation and limited knowledge	Misinformation spread through word of mouth Lack of individual and community knowledge around this issue
The costs of purchasing safer hair products	Natural and safe products are more expensive Large time investment when shopping for and using safer products
Underlying concepts	
Distrust in government and large corporations	Driven by the lack of regulations/policies and connected to a history of racism Driven by lack of transparency in terms of ingredients and profit-driven nature of companies
Individual burden/responsibility to shop for safer products	Connected to distrust in government and large companies Shopping for safer products is one of many responsibilities to navigate
Facilitators	
Back to our cultural and community roots	Back to our cultural roots (word of mouth) Back to the basics (do-it-yourself products) Back to our natural roots (transitioning back to natural hair)
Raising individual knowledge and public awareness for action	Research process to learn about safer products: word of mouth and internet searches Preventative behaviors to reduce exposure

### Barriers to safer hair product purchasing and use

The five barrier themes spanned several levels and domains of influence (Figure 1). These themes included (1) unsafe ingredients allowed in US products, (2) differences in access to safer hair products, (3) style (product design and branding) over substance (ingredients), (4) the costs of purchasing safer hair products, and (5) navigating misinformation and limited knowledge.

### Unsafe ingredients allowed in US products

Several co-researchers highlighted the lack of US government regulation regarding food, personal care, and consumer products. They identified unsafe chemicals or ingredients that are allowed in products sold in the US including EDCs (commonly referred to as “endocrine blockers” by the co-researchers) and preservatives in personal care products and hormones in food. These discussions illustrated that the co-researchers view personal care products as one of several consumer product categories where unsafe ingredients are found. Several co-researchers also discussed the differences in the regulatory landscape between the US and other countries.

*“Some of the oils and some of the products that are in America are not, you know, available in Europe. My husband is from Europe, and we spend a lot of time in Europe and I see the difference and you know, sometimes it’s hard for me not to stop and think about what if we use different products in America and products that could benefit us…but uh, it’s just the way it is.”* JB (Note: co-researchers had the choice of using identifiers, such as their initials, letter of their first name, or to remain anonymous).

### Differences in access to safer hair products

Most co-researchers reported experiencing differences in access to safer and more natural hair products between neighborhoods in Boston, MA. The products in their communities were described as *“junk,”* containing *“loads and loads of stuff,”* and *“cheap…and gonna harm [us] in the long run”* while products in higher-income White communities were described as *“more precise,” “higher quality,” “better products…more of it to choose from.”* These experiences were a common sentiment shared across groups, however, there was nuance in the discussion and a few co-researchers in one group (G1) reported safer hair products were available in their community, however, the time to search for them was an additional barrier. Others (G3) reflected that while communities of color had more beauty supply stores (indicating increased access to hair products overall) there was decreased access to safer hair products.

*“You can live in Roxbury I’m just gonna throw that out there if they are inexpensive, they just give you any old thing. They put it out there for people to buy it, because they feel like everybody’s poor so they’ll buy these cheap things and it’s gonna- not knowing it’s gonna harm them in the long run. But when you go out to the suburbs some of those places have better products, they have a lot more of it to choose from and you know you never know what’s going on, and they are not trying to [rob] you. Well, some of them are, but you know it’s more geared towards the, I’ll say the [urban] part of the cities than it is towards the suburb part of the cities.”* DC.

Differences in access to safer hair products were also observed within stores through physical barriers. Several co-researchers noted that products marketed to or used by communities of color were locked up behind plexiglass creating an additional challenge to purchasing hair products or reviewing labels. Additionally, one co-researcher noted a theft sensor placed on top of the ingredient label which blocked her ability to review the ingredients.

### Style (product design and branding) over substance (ingredients)

The role of product design and branding as barriers to safer product use was discussed by many of the co-researchers. Specifically, products were believed to be designed to attract potential customers through bright colors, large fonts for certain names/words, and catchy phrases with the product’s “active promise” (such as Doo Gro, Triple Strength, etc.). In comparison, several co-researchers noted the ingredient labels were smaller and difficult to read. Several also discussed the use of uncommon ingredient names on labels when there are more common names.

*“I also think about just the marketing practices of making print so small. I mean like others are saying we were jumping through hoops just to figure out what we are putting on our hair, you know, we are attracted to all the glitz and the color and the big you know the big things we get there.”* AP.

In addition to the design/packaging, the co-researchers reported product branding/marketing also contributed to challenges in shopping for safer hair products. Specifically, some co-researchers spoke about the fact that organic is only used for branding and is a “buzzword.”

### The costs of purchasing safer hair products

Two main costs were identified as barriers: price and time. Most co-researchers highlighted how safer and more natural products were more expensive and discussed the challenges of purchasing safer products within their price point.

*“We are all living in this very, very harsh economy and what we are looking for it can be out of our price point, and if we are not able to afford the stuff that we need, especially not just, you know, hair products and body care products, but also food, you know we are going to turn to the cheapest, most dangerous option that is in order to supply a need that we have. And my research was, I think it took me like a week to even find something that came close to minimum [endocrine] disruptors and you know something that came close to “natural” and this was it that was affordable. Everything else was like $50.00 for the shampoo and $30.00 for the conditioner.”* T.

**Figure 2:**
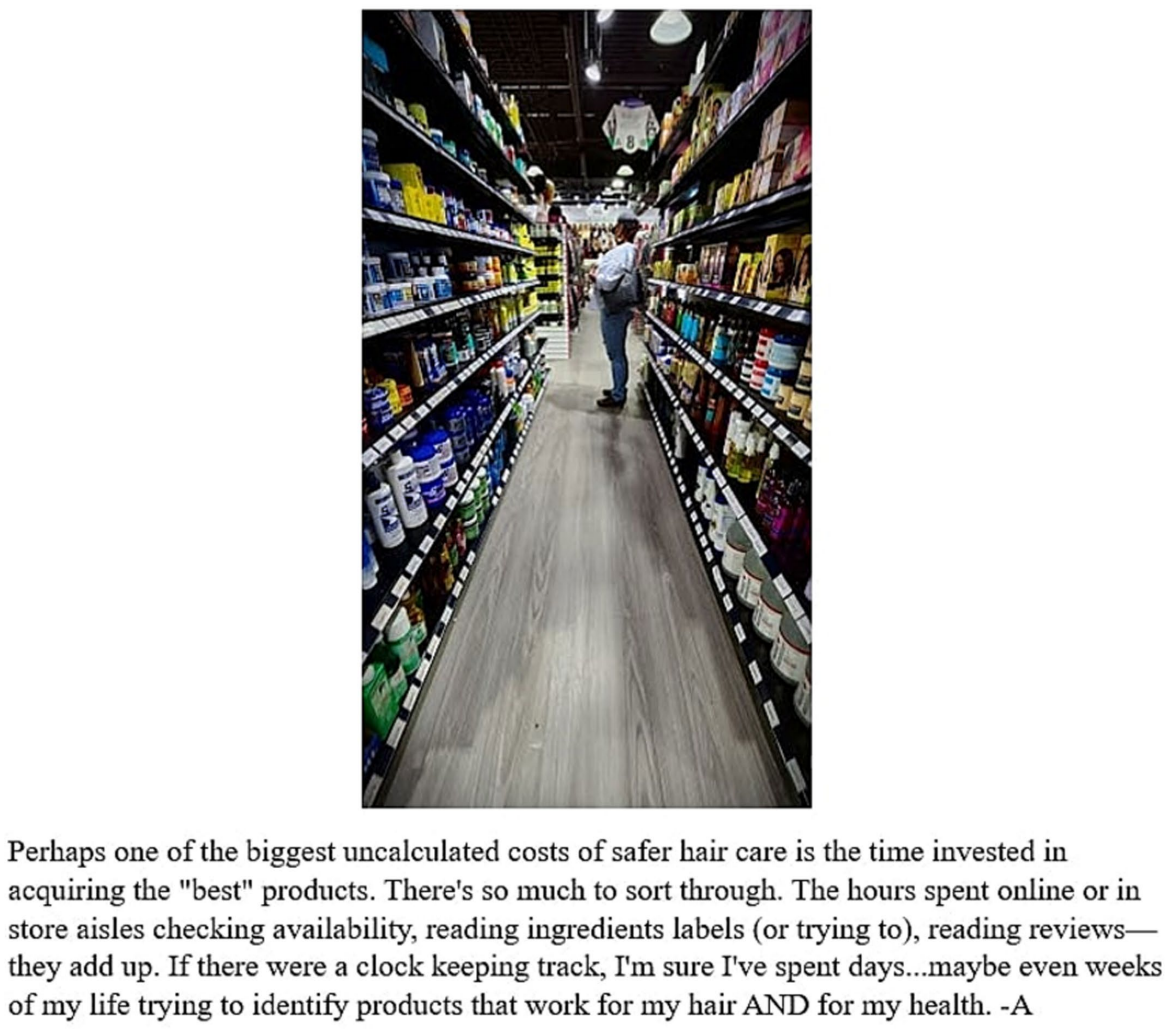
Photo taken by a co-researcher titled “Overwhelm” and photo caption.

Additionally, the co-researchers spoke about a variety of dimensions related to time as a barrier to safer hair product use. This idea was exemplified by one co-researcher, A, who stated in a photo-elicitation interview how “*it’s time across the experience, right, of finding, understanding, actual acquisition of a product, use of a product, you know, I think for a lot of women, especially Black and brown women, there’s also still that trial period*” (Figure 2). Several co-researchers noted the lack of time they have to go through the process of purchasing safer hair products which drives them to rely on other assessments, such as price or word of mouth.

*“It’s a little mini job to find a time like I would love to have the time to, you know, research products. Like, I already spend a lot of time working, taking care of my two kids, then trying to take care of the house and, you know, the many other things that we all have to do that taking that active role of self-care when it comes to our hair, you know, we are like, should I prioritize my hair? Should I prioritize what I eat? Should I prioritize exercising? Like which one should I prioritize? So sometimes you know, we have to choose, and our hair may not make the cut. So, we end up not putting in the time that we would like to.”* PP.

### Navigating misinformation and limited knowledge

Many co-researchers identified misinformation—incorrect or misleading information—and limited knowledge as barriers to using safer hair products. Word of mouth was noted as a common avenue through which misinformation was spread. A few of the co-researchers discussed experiences of individuals within their social networks sharing information regarding harmful ingredients that conflicted with their research or information shared by the RESTYLE team.

*“You know you got scientists saying something like the tea tree is an endocrine blocker. Then you got it this one [name of local business owner] saying, no, if you just have less or know your body is not to be allergic to the item, then it will not be an endocrine blocker. Who knows?”* LM.

Furthermore, a couple of co-researchers shared incorrect information during the focus groups about the safety of placenta-containing products and the requirement of testing for personal care products before entering the market. Some co-researchers believed that products containing placenta and essential oils are safe to use since those ingredients are viewed as natural. In the context of a lack of accurate information, many co-researchers across groups spoke about the importance of education.

Additionally, many co-researchers acknowledged the lack of individual and community knowledge regarding this issue. As an example, across several focus groups, co-researchers described how they learned through the initial training that lavender and tea tree essential oil may have endocrine disrupting properties, however, this information is not widespread among their community who are frequent users of these products.

*“So, people need to be aware of this is not good for you. Like who would know, tea tree oil. Do you know how many people use that? So many people, there’s, like, unbelievable across the board because we see tea tree we are thinking healthy. Oh, I can definitely use that, that’s one of the best oils there is, it’s good for you. We’re literally in the line thinking that is something good just to find out that it’s something bad and it’s something that we should not be using but do you know how many, how many people in America in this world actually do, because no one knows—there is no awareness when it comes down to, you know, the health of your body, when it comes down to, you know things that we wash our body with, things we wash our hair with.”* TJ in a photo-elicitation interview.

### Underlying concepts

Two underlying concepts were identified across the barriers: Distrust in government and large corporations and individual burden/responsibility to shop for safer products (Figure 1). Several co-researchers discussed that while the government is supposed to protect the public, they are not fulfilling their duties due to the lack of laws and regulations. Additionally, a few co-researchers alluded to the history of discrimination and racism in the US tied to current-day distrust in government and institutions. The following quotes from different groups (G2 and G3) reflect these sentiments:

*“I just do not understand our government also because you know the FDA [Food and Drug Administration] and all that we are paying all this tax paying money for them to keep us safe with food and products and they are dropping the ball at all costs mean like it’s like the wolf minding the hen house and it’s just tragic, it’s tragic and it has a history in America anyway, you know what I mean, experimentation on us or what have you and here it is in 2023 we cannot even buy a product that would keep us healthy. So, the government is definitely dropping the ball.”* LM.

*“The government does not watch any of these companies on what they do or what they put in these products, especially when they are selling to the urban areas. They do not care, they have been, I say it again, they have been doing everything in their power to kill off the people with melanin and we are still here, no matter what you do, you are not gonna like we are not going anywhere. So, they can sit here and keep trying. They’re gonna keep trying, this is why we are sick all the time. This is why all these diseases keep coming up because they keep putting these products, these chemicals in our products in our foods and we keep getting sick, and all these cancers and stuff. It’s like a cycle, it’s a cycle in America.”* MM.

The lack of transparency regarding ingredients and the profit-driven nature of large corporations contributed to the co-researchers’ distrust of large companies. Specifically, many co-researchers questioned why there were differences in access to safer products and “hidden” or unsafe ingredients in their products. They also questioned the practice of small Black-owned brands being purchased by large “White” corporations—this sentiment was exemplified by KL.

*“In the urban community, many African and migrant women voiced their concerns and experience with the beauty products after using it within a year. For a product that makes our hair grow healthy and feel rich causes us so many damages after certain time frame. The loss of our hair, skin irritations and loss of confidence it come to realization that the black owned hair products is sold by a white man which they change the hair ingredients. They even try to use the words “natural, organic, healthy, safe” as part of their marketing. Now many urban community started to make their own homemade natural products from home. Not sure why the white man is so focused on the black brand and black hair, what can they possibly know about?!? The real truth is explained in the movie called “They cloned Tyrone” also many of the hair products are going through lawsuits due to cancer. Only the black community is affected by this.”* - KL.

Profits were identified by several co-researchers as the main motivator behind business decisions.

*“I feel as if in the urban community we find the products that really works for us and after a year we are noticing that the products is not working for us. I noticed that the products either making us lose our hair, having like skin irritation and for many years even like hearing my mom and grandmother talk about it like oh, I used to buy this hair product, it used to work, it made my hair grow and now it does not work anymore blah blah and I noticed that because a lot of this black businesses like they’ll sell it to the corporate, meaning they will sell it to the White people and once the White people get their hands on it, they’ll start changing their ingredients, which I do not know why. They always have to be in Black people’s business.”* KL.

Another concept underlying the aforementioned themes was that the onus is currently placed on individuals to shop for safer hair products. Many co-researchers expressed this sentiment and discussed how this was driven by the lack of government regulations and transparency among businesses emphasizing the connection to the previous underlying concept of distrust.

*“I think it kind of underscores how this is kind of like the wild wild West, you know I’m not sure exactly what is safe and they are not that many regulations and I think, unfortunately, it’s really up to us as consumers to try to do as much as we can within limitations such as price, you know, how far we are gonna do research, how far we are gonna go out to travel to get something and so I think that unfortunately it’s a lot on us as consumers.”* AO.

Furthermore, several co-researchers expressed that the individual burden to shop for safer products was only one of many responsibilities and priorities they navigated daily (connecting back to the time cost). The following quote summarizes a shared sentiment regarding the desire to more easily purchase safer products:

*“Life can be so complicated, it would be nice just to be able to pick up anything and use anything and not have to worry about the impact on your skin or on your children or on the planet. But the fact of the business is that’s a luxury that we really do not have anymore.”* Anonymous (A) 2.

### Facilitators to safer hair product purchasing and use

The two themes focused on facilitators to safer hair product purchasing and use were noted across the community, interpersonal, and individual levels of influence (Figure 1). The themes were (1) going back to our cultural and community roots and (2) raising individual knowledge and public awareness for action.

### Going back to our cultural and community roots

Co-researchers across groups discussed the importance of going back to their community and cultural traditions in terms of safer hair product use. This multidimensional theme encompassed the importance of word of mouth in sharing information (back to our cultural roots), using DIY products (back to the basics), and embracing our natural hair texture (back to our hair roots). Many co-researchers discussed the community and cultural importance of sharing information, resources, and recommendations via word of mouth. This desire to share information exemplified their communities’ history of oral traditions that differ from the “dominant culture” which was viewed as individualistic by A2 and agreed upon by others. In practice, these oral traditions occurred through sharing recipes, recommendations for products, news/information about hair product safety, and reaching out to friends and family members for advice about ingredients or products.

**Figure 3:**
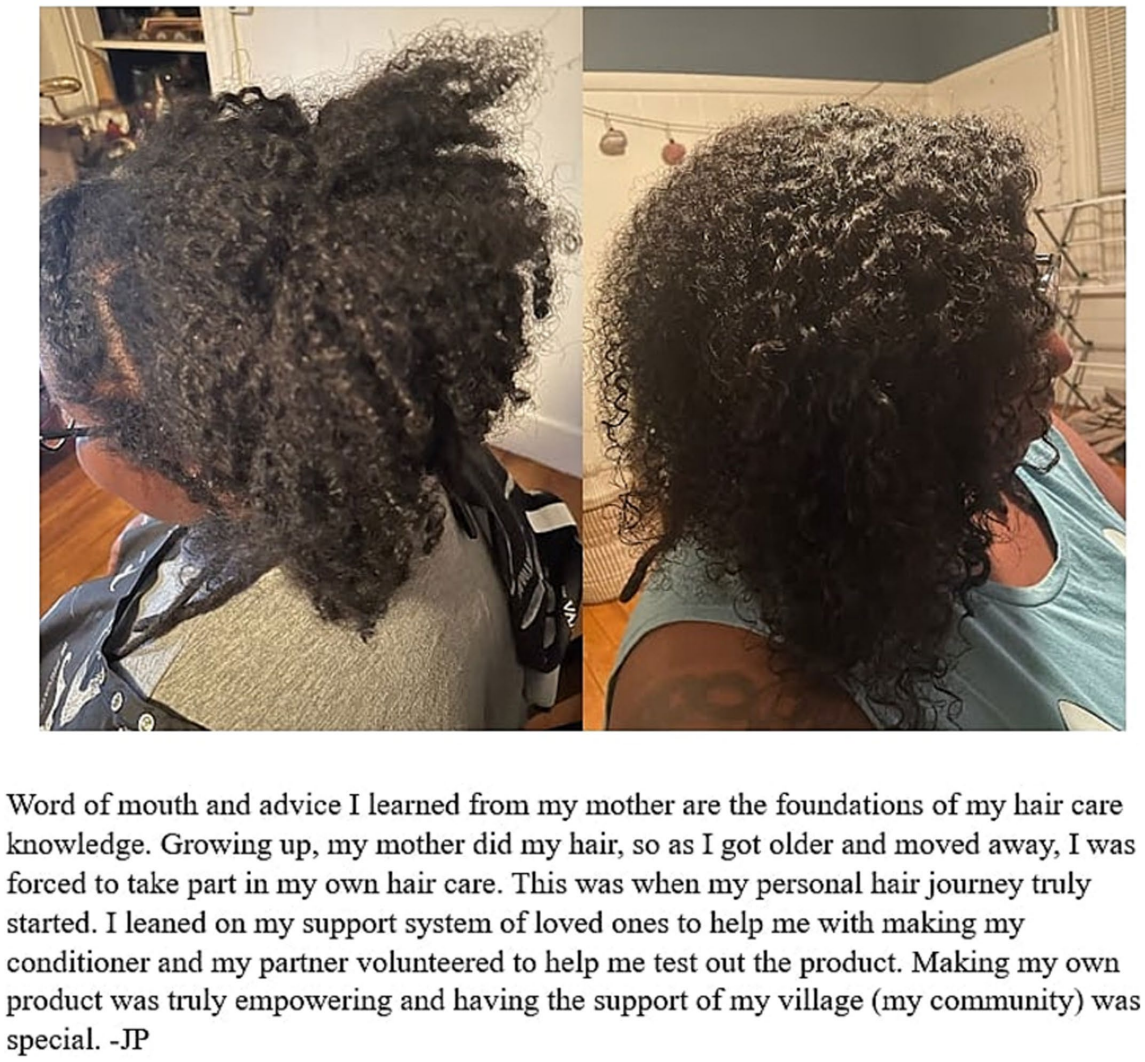
Photo taken by a co-researcher titled “Natural Village Conditioner” and photo caption.

*“Just talking to your friends and families more about what you have learned. For me, this was new it wasn’t like—I know a lot of people in this group mentioned they have had some experience in the past with all this stuff, so they are familiar with the terms and all that. But for me it was pretty new so um I now can go to my friends and share what I’ve learned. My roommates, for example, were helping me make my products, so like now they know, now they know what to look for on the bottles, so just have more conversations with your friends and families and just pass on that knowledge that you have learned that’s what I plan on doing anyway.”* JP (Figure 3).

One common recommendation related to using safer products was DIY products. Several co-researchers highlighted the modern reliance on store-purchased products, while historically their ancestors and families created products from the earth. Furthermore, many viewed DIY products as symbols of a collective experience, tying back to the importance of community. These products were also viewed by many co-researchers as cheaper than store-bought products.

A shared experience among the majority of co-researchers was the use of chemical hair relaxers when they were younger and eventually transitioning to natural hair. This process of embracing their natural beauty and hair texture was discussed by several of the co-researchers (mainly G1 and several interviews). Specifically, by embracing their hair texture and wearing their hair shorter—they used less chemical hair relaxers. In a photo-elicitation interview, A1 discussed her transition to natural hair and the process of embracing her hair texture:

*“[I am] self-conscious and self-aware that my hair is short so I feel like yeah, sometimes I feel like the world would judge me because I have short hair. And I’m starting to come out of that shell like, who cares what people think? Either you are gonna like me or you are not gonna like me and I’m at the point where I do not really care if you like me or not because it should not be about my hair. It should be about who me, as a person that you like, not what my hair looks like.”* A1.

### Raising individual knowledge and public awareness for action

The majority of the co-researchers discussed their research process for learning about products which consisted of a combination of word of mouth for initial recommendations followed by using social media and internet search engines to learn more about these products or ingredients. The most common online sources for information were Google and YouTube—others included TikTok, Amazon, Instagram, and Facebook. Research was an important part of their process of selecting hair products and an opportunity to increase their knowledge regarding safer products.

Many of the co-researchers had some prior knowledge that hair products may contain unsafe ingredients (while fewer knew about specific ingredients such as EDCs before the initial training). Based on this knowledge they adopted preventative behaviors, such as searching for shorter ingredient labels or more common/easily understood words, to attempt to limit their exposure to chemicals of concern. While these preventative behaviors may have protected them from exposure to some chemicals of concern, other ingredients that are common/easily understood and used by this community—such as essential oils—are reported to have endocrine disrupting properties. Additionally, after the initial training—which discussed how certain essential oils are potential EDCs and differences in exposure based on the use of leave-in versus rinse-out products—a couple of the co-researchers considered alternative preventative behaviors.

*“So, I’ve decided I’m going to use that [tea tree shampoo and conditioner] because those are rinse-out products and then one of the focus groups there was made mention of how…it might not be best to leave in your hair as opposed to use as a treatment and rinse out.”* A2.

Furthermore, increased public awareness surrounding this issue was identified by some of the co-researchers. Specifically, one facet of public awareness that was highlighted across groups was knowledge of the Johnson & Johnson lawsuit related to the presence of asbestos in talcum powder used in personal care products (31). This increased public awareness, resulted in preventative behaviors and a couple of co-researchers discussed how their families or themselves no longer use Johnson & Johnson products.

## Discussion

In this analysis, we used photovoice, a qualitative community-based participatory method to identify the barriers and facilitators to safer hair product purchasing and use among Black women in the greater Boston, MA area. We identified themes related to barriers ranging from the societal level (unsafe ingredients allowed in US products) to the individual level (the costs of purchasing safer hair products). Concepts underlying the barriers were distrust in government and large corporations and the individual burden/responsibility to shop for safer products. Furthermore, we identified two themes related to facilitators that were rooted in the individual to community level: going back to our cultural and community roots and raising individual knowledge and public awareness for action.

Previous qualitative research has explored the experiences and perceptions of personal care product use among diverse individuals (18, 19). A community-based analysis from Vilfranc et al. explored the hair journeys of women of color in Northern Manhattan through focus groups and discussed themes including products that impacted their hair journey, factors (including people or entities) that shaped hair experiences, and the relationship between hair and “sense of self” (18). Additionally, an analysis in Southern California by Teteh et al. conducted key informant interviews and focus groups among Black men to explore knowledge and perceptions regarding Black women’s hair, product use, and breast cancer risk factors (19). The themes identified included perceptions of Black women’s hairstyles, lack of knowledge regarding the potential effects of hair product use and cancer risk, and the importance of educating the community about potential risks associated with hair product use and cancer. Social pressures and beauty standards were discussed in both analyses as potential drivers of hair product use patterns. This differed from our analysis where we noted embracing hair texture as a facilitator but not beauty standards/societal pressure as a barrier. We hypothesize our findings to be driven by the fact that many of the co-researchers had already transitioned from the use of chemical hair relaxers to natural hair. This process of transitioning coincided with embracing their natural beauty and hair texture, thus, for the co-researchers, these social pressures may not currently be barriers to safer hair product use.

Our study reported time and price to be notable barriers to safer hair product use among the co-researchers. Dodson et al. examined personal care product use patterns among diverse women in California and also reported cost as a main consideration when choosing products (7). Furthermore, other research in the context of hair maintenance and physical activity among Black women has reported substantial financial and time investments (32–34). Thus, Black women may be experiencing hair product-associated time and financial burdens across several areas related to health (e.g., physical activity and personal care products).

A shared sentiment among the co-researchers was how products were designed/marketed to attract consumers while less effort was perceived towards ingredient transparency. This contributed to the feelings of distrust towards large corporations. Specifically, the co-researchers believed organic to be a marketing term. These sentiments may illustrate greenwashing—when a company presents misleading information or disinformation about a product, program, or actions as environmentally or socially responsible (35). The use of these terms to market products may be driven by the increased consumer demand for sustainable, natural, and organic products in recent years (36–38). However, research has demonstrated that consumer awareness of greenwashing or “green skepticism” results in decreased consumer satisfaction due to belief in “corporate hypocrisy” (39). For the co-researchers, this concept may be actualized through distrust of large corporations. Future research should further explore how business decisions and marketing/branding may be barriers to safer personal care product use to inform future interventions.

Differences in access to safer hair products between neighborhoods and within stores were observed by the co-researchers. These experiences supported our previous quantitative research identifying differences in the safety of hair products between sociodemographically diverse neighborhoods in Boston, MA with lower income and/or communities of color having the highest risk (40). Through the current analysis, we provided additional context to these findings by illustrating that community members have noted differences in hair product quality in their neighborhoods compared to others. Furthermore, the co-researchers reported physical in-store barriers of plexiglass and theft sensors on labels of products used by communities of color. This may be another example of retail redlining, a discriminatory practice of not serving or underserving certain communities based on the sociodemographic composition of customers (41). Future research should examine these practices in stores and how they may contribute to differences in the purchasing and use of hair products.

Within the theme of going back to our roots, we identified a combination of factors that supported safer product use including word of mouth from family/friends, DIY products, and embracing natural hair texture. The aforementioned analyses from Dodson et al. and Vilfranc et al. reported that family members had an impact on individuals’ hair decisions (7, 18). In our analysis, word of mouth from social networks was also identified as a barrier based on spreading misinformation. This finding emphasizes the importance of future interventions focused on education—a solution highlighted by many co-researchers. In terms of DIY products, there is limited research exploring the drivers and practices of creating DIY products among communities of color. However, James-Todd et al. evaluated the use of “other hair products”—including oils, eggs, and grocery items—and reported higher ever use among African American and Afro-Caribbean women compared to other racial/ethnic groups (6). Future research should explore how the creation of DIY products may support safer product use among this community.

Additionally, increased awareness of chemicals of concern and associated health effects may have contributed to the implementation of protective behaviors among the co-researchers. These concepts may speak to the co-researchers’ environmental health literacy (EHL)—how information on environmental health is used to make informed decisions and influence behaviors (42–44). In the context of personal care product chemicals, a previous analysis from Tomsho et al. developed an EHL scale related to phthalates (a personal care product EDC) and identified factors related to awareness of chemicals of concern, exposure pathways, health effects, protective behaviors, and general knowledge (42). While exploring EHL was outside the scope of this project, co-researchers demonstrated increased awareness of chemicals of concern in addition to new or longstanding protective behaviors.

This analysis has several limitations. First, RESTYLE co-researchers were recruited through community partners, some of which are focused on improving community health. Thus, based on this and their prior knowledge of chemicals of concern and associated health outcomes the co-researchers may have increased EHL regarding this issue which may result in different barriers/facilitators compared to other community members. Second, our co-researchers were between the ages of 30–70 and the majority have transitioned to natural hair—therefore, our results may not reflect the experiences or views of younger or older individuals or individuals who currently use chemical hair relaxers. Third, we recruited Black individuals with English proficiency based on our study team’s linguistic abilities. Based on these reasons and our focus on the greater Boston area, our results may not be transferable to other communities of Black women in the US. Fourth, all study activities were virtual based on the co-researchers’ preferences. Thus, while this may have allowed the co-researchers to attend focus groups that they would not have been able to in person due to scheduling, there may be different interpersonal dynamics in virtual versus in-person focus groups. Fifth, based on privacy concerns regarding naming and taking photographs of certain brands and products, the co-researchers were not invited to join us as co-authors. However, for our future analyses reporting back on potential interventions and solutions we plan to invite all co-researchers to join us as co-authors. Lastly, qualitative analyses are subjective, however, we aimed to reduce bias through a variety of tools to ensure credibility and dependability/confirmability (prolonged engagement, investigator triangulation, and an audit trail).

This analysis also has several strengths. This study aimed to explore factors related to safer hair product purchasing and use, considering factors at the individual level, as well as more upstream. Identifying upstream factors may contribute to the development of interventions and solutions to this issue that reduce the individual onus discussed by the co-researchers. Next, our analysis specifically focused on the barriers and facilitators to safer hair product use to inform future research, interventions, and solutions. This adds to previous research that has predominately focused on identifying inequities in exposure and differences in personal care product use patterns. Future research among the RESTYLE study will use these results to inform the identification and development of interventions and solutions. We used photovoice, a community-based participatory qualitative method that aims to empower co-researchers to take action regarding the issues and resources within their community. To our knowledge, our analysis is one of the first to use photovoice to explore inequities in EDC exposure from hair product use. Throughout the process, we noted the co-researchers discussing what they learned in the initial training on personal care product safety as well as potential preventative behaviors they may take. The co-researchers also highlighted the importance of sharing this information with others in their network. Lastly, many co-researchers wanted to continue with RESTYLE and engage in future trainings, workshops, and/or research. Thus, while not quantified, we illustrate that through this process the RESTYLE co-researchers may have gained knowledge and be empowered to make informed decisions and take action regarding this issue.

Overall, this analysis identified several barriers and facilitators to safer hair product purchasing and use among Black women in the greater Boston area through photovoice. The barriers included a lack of government regulations, differences in access to safer products, product design/branding, as well as individual factors—illustrating the importance of a multipronged approach to interventions. In comparison, the facilitators were focused on increasing knowledge and highlighted the importance of staying rooted in their community to find safer products. Hair product use is a modifiable risk factor that may be associated with a variety of health outcomes across the life course where inequities persist (6, 14). Future research should continue to explore the barriers and facilitators to safer product use among diverse communities and work to develop interventions and solutions.

## Data Availability

The datasets presented in this article are not readily available because we conducted qualitative research that shared the stories and experiences of community members. Even though the data is anonymized or uses brief personal identifiers (i.e., initials), based on the stories and experiences shared and community partners we worked with the transcripts can never fully be anonymous. Requests to access the datasets should be directed to Marissa Chan, marissachan@hsph.harvard.edu.
