# Supplementary material for: Exploring the barriers and facilitators to safer hair product purchasing and use among Black women in the greater Boston area through photovoice in the RESTYLE study
Source: Front Public Health. 2025 Aug 18;13:1513671. doi: 10.3389/fpubh.2025.1513671 (PMC12401100; doi:10.3389/fpubh.2025.1513671)
Supplement: Supplementary file 1 [file Supplementary_file_1.docx]

**SUPPLEMENTAL MATERIALS**

**Supplemental Table S1:** RESTYLE partners and description

| Partner | Description | New or Existing Partnership |
| --- | --- | --- |
| Bethel AME Church, Jamaica Plain | A local congregation started by two medical doctors in Boston; connected to the church, the Bethel Institute for Social Justice works to fund community-based academic and social service programs aimed at supporting the marginalized communities in the greater Boston area. | Existing; our ongoing collaborations include an annual youth educational summer camp and educational workshops focused on topics such as personal care product chemicals, access to healthy foods, and brain health. |
| Union Capital Boston | An organization that transforms social capital and relationship building through rewarding community engagement and creating networking opportunities. | New |
| Resilient Sisterhood Project | A Boston-based community organization focused on educating and empowering Black women and girls about reproductive health. | Existing; we have previously partnered with them on community-engaged research focused on heavy metals exposure among communities in Boston. |
| Southern Jamaica Plain Health Center | A community health center licensed by Brigham and Women’s Hospital that services Jamaica Plain residents and surrounding communities. | New |
| Comics in Color | An organization and festival created to develop a space for people of color who create/enjoy comics and more broadly art in Boston. | New |

**SUPPLEMENTAL FIGURE**

**
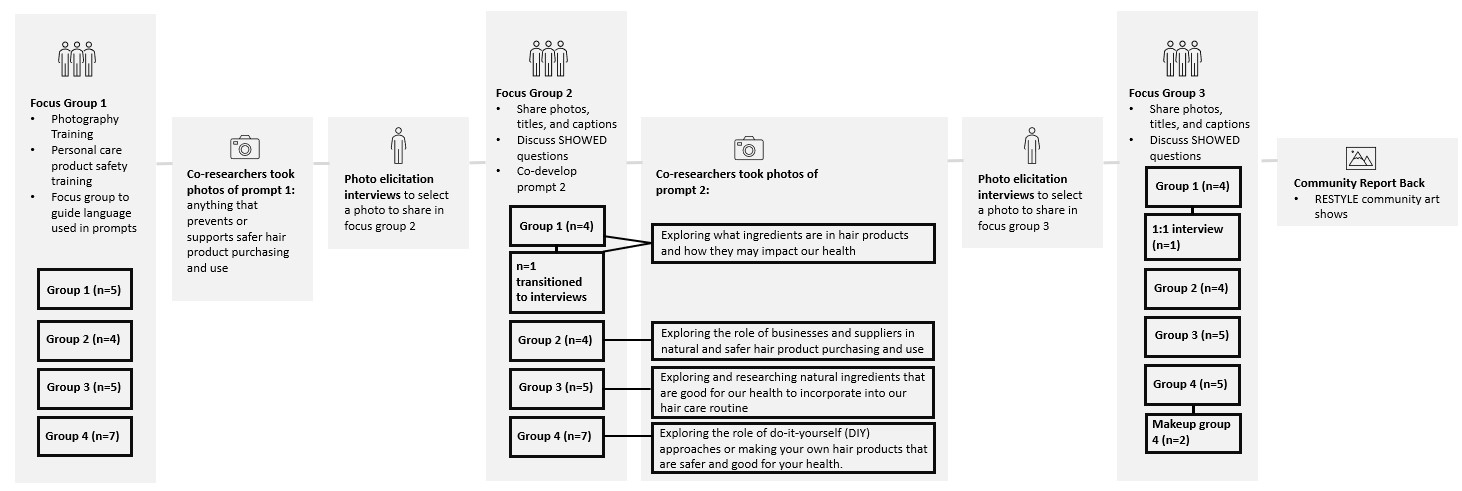
Supplemental Figure S1:** RESTYLE photovoice process
